# Phenotypic and Functional Changes in Peripheral Blood Natural Killer Cells in Crohn Disease Patients

**DOI:** 10.1155/2020/6401969

**Published:** 2020-02-19

**Authors:** Suzanne Samarani, Patrick Sagala, Prevost Jantchou, Guy Grimard, Christophe Faure, Colette Deslandres, Devendra K. Amre, Ali Ahmad

**Affiliations:** ^1^Laboratory of Innate Immunity, University of Montreal, Montreal, Quebec, Canada; ^2^CHU Sainte-Justine Research Center, University of Montreal, Montreal, Quebec, Canada; ^3^Department of Microbiology & Immunology, University of Montreal, Montreal, Quebec, Canada; ^4^University of Montreal, Montreal, Quebec, Canada; ^5^Department of Pediatrics, University of Montreal, Montreal, Quebec, Canada; ^6^Department of Surgery, University of Montreal, Montreal, Quebec, Canada

## Abstract

We investigated activation status, cytotoxic potential, and gut homing ability of the peripheral blood Natural Killer (NK) cells in Crohn disease (CD) patients. For this purpose, we compared the expression of different activating and inhibitory receptors (KIR and non-KIR) and integrins on NK cells as well as their recent degranulation history between the patients and age-matched healthy controls. The study was conducted using freshly obtained peripheral blood samples from the study participants. Multiple color flow cytometry was used for these determinations. Our results show that NK cells from treatment-naïve CD patients expressed higher levels of activating KIR as well as other non-KIR activating receptors vis-à-vis healthy controls. They also showed increased frequencies of the cells expressing these receptors. The expression of several KIR and non-KIR inhibitory receptors tended to decrease compared with the cells from healthy donors. NK cells from the patients also expressed increased levels of different gut-homing integrin molecules and showed a history of increased recent degranulation events both constitutively and in response to their in vitro stimulation. Furthermore, treatment of the patients tended to reverse these NK cell changes. Our results demonstrate unequivocally, for the first time, that peripheral blood NK cells in treatment-naïve CD patients are more activated and are more poised to migrate to the gut compared to their counterpart cells from healthy individuals. Moreover, they show that treatment of the patients tends to normalize their NK cells. The results suggest that NK cells are very likely to play a role in the immunopathogenesis of Crohn disease.

## 1. Introduction

Natural Killer (NK) cells are important effector cells of the innate immune system. They comprise about 10-15% of the mononuclear cells in the peripheral blood [1–3]. Phenotypically, they are non-T and non-B lymphocytes and express CD16 (Fc*γ*RIIIa; the low-affinity receptor for the Fc region of IgG) and CD56 (an isoform of the Neural Cell Adhesion Molecule (N-CAM)) on their surface. Based upon the levels of expression of these two molecules, NK cells have been subdivided into different subsets of which CD56^bright^CD16^−^ and CD56^dim^CD16^+^ have been well characterized [4]. NK cells can target and kill autologous cells of the body when the latter become infected with intracellular pathogens (e.g., viruses) and are transformed or stressed due to DNA damage and/or inflammatory stimuli. They can also kill and eliminate target cells with the help of antibodies in a process called antibody-dependent cell-mediated cytotoxicity (ADCC) [3, 5]. The cells targeted in ADCC express specific antigens for the antibodies, which bind to CD16 on NK cells. In conjunction with autoantibodies, NK cells can promote autoimmune diseases [6] (reviewed in [7]). Furthermore, they can also regulate immune responses by secreting a variety of soluble mediators such as IFN-*γ*, TNF-*α*, GM-CSF, MIP-1*α*, MIP-1*β*, and RANTES [8]. By secreting these soluble mediators, NK cells can affect the quality and strength of an adaptive immune response. They can also modulate adaptive immune responses by their interactions with dendritic cells (DC), effector T cells, and Tregs [9, 10].

NK cells express a wide variety of inhibitory and activating receptors as well as costimulatory molecules on their surface [3, 11, 12]. The receptors and coreceptors bind to their cognate ligands on the surface of target cells. The balance of inhibitory and activating signals received by an NK cell determines whether the target cell in question will be killed or spared. From the functional point of view, the most important of these receptors are Killer-cell Immunoglobulin-like Receptors (KIRs), which are either of inhibitory or activating type [12–14]. Each KIR, especially in the case of inhibitory ones, binds to public epitopes present on a subset of related MHC class I/HLA molecules. Different inhibitory KIRs bind with their cognate HLA ligands with different affinities and exert varying degrees of inhibition on NK cells. KIR receptors are encoded by the same-name (*KIR*) genes. The *KIR* gene family is polygenic and highly polymorphic. The individuals that inherit KIR-HLA genotypes that exert relatively weaker inhibition of their NK cells and/or inherit an increased number of activating *KIR* genes present relatively more resistance to intracellular pathogens. They can control and clear viral and microbial infections relatively more efficiently as compared to the individuals who inherit KIR-HLA genotypes that exert tighter inhibition of their NK cells and/or inherit none or a smaller number of functional activating *KIR* genes [13, 14]. Such individuals are also more resistant to the development of a variety of cancers. However, they are more prone to the development of different autoimmune and chronic inflammatory diseases. In this regard, inheritance of less inhibitory KIR-HLA genotypes and a higher number of activating *KIR* genes has been associated with the development of several autoimmune diseases such as ankylosing spondylitis, type 1 diabetes (T1D), multiple sclerosis, and rheumatoid arthritis [13, 15–17]. It has been proposed that NK cells in these individuals have a relatively low activation threshold, become activated from different environmental triggers, cause autoaggression, and promote inflammation. Consistent with this theme, we have recently shown significant positive associations of activating KIR genes with the development of Crohn disease (CD) using three independent cohorts of Caucasian CD patients [18]. CD is a chronic inflammatory disease of the gastrointestinal tract that particularly affects the terminal part of the ilium and colon. The disease has also an autoimmune component as the patients develop a variety of autoantibodies that target antigens such as pancreatic autoantigens, e.g., glycoprotein-2, CUB and zona pellucida-like domain-containing protein 1, GM-CSF, and phospholipids [19–22]. The significant positive association of activating KIR genes with CD suggests involvement of NK cells in the immunopathogenesis of this disease. It also suggests that NK cells from the patients may express these receptors at higher frequencies and consequently may exhibit lower activation thresholds. Furthermore, NK cells may also become overactivated and cause autoaggression under inflammatory and autoimmune conditions [23, 24].

We hypothesized that NK cells from CD patients are in a higher activation state and are more cytotoxic compared with the cells from healthy subjects. To test this hypothesis, we investigated the expression of different activating and inhibitory receptors, gut-homing integrins, activation status, and recent degranulation history/cytotoxic potential of the peripheral blood NK cells in CD patients and compared them with those from healthy controls. The results are reported in this research article.

## 2. Materials and Methods

### 2.1. Study Population

For these studies, whole peripheral blood from twenty-one CD patients and twenty healthy controls was used. All the study participants were 6-17 years of age and were Caucasians residing in the province of Quebec (Canada). Of the CD patients, nine were newly diagnosed, six had relapsing disease, and six were in remission. The newly diagnosed and relapsing patients were not receiving any treatment at the time of blood sample collection and were treatment-naïve. The patients in remission were currently receiving TNF-*α* blocking antibodies (infliximab or adalimumab), prednisone, or azathioprine. They are considered treated patients. The demographic and clinical parameters of the study participants are given in Table 1. Controls (20) were age- and sex-matched donors who came to Ste-Justine Hospital as visitors and had no known disease. Written informed consent was obtained from all the blood donors or their legal guardians. The study was approved by the Research Ethics Committee (comite d' éthique de recherche (CER)) of CHU Ste-Justine.

### 2.2. Flow Cytometry

For determining different NK cell subsets (with respect to their expression of CD16 and CD56), whole peripheral blood from each donor was used. Before staining, Fc Receptors (FcR) were blocked in 1 ml of whole blood using the Human FcR Binding Inhibitor (eBioscience, catalog #14-9161-73). RBCs in the samples were lysed using RBC lysis buffer from eBioscience (catalog #00-4300-54). After washing with PBS containing 0.5% FBS (PBS/FBS), aliquots (100 *μ*l each) were stained with PerCp-eFluor 710-conjugated anti-human CD3, APC-Cy7-conjugated anti-human CD14 and CD19, APC-conjugated anti-human CD56, and Pe-Cy7-conjugated anti-human CD16 antibodies (see Table 2 for vendors and catalog # for the antibodies used in this study). NK cells were gated and their subsets determined as shown in Supplementary [Supplementary-material supplementary-material-1].

The expression of KIR, non-KIR receptors, activation markers, and integrins was determined on CD3^−^CD14^−^CD19^−^ NK cells, which were stained with fluorochrome- (PE or FITC) conjugated marker-specific monoclonal antibodies (listed in Table 2). Only for one receptor (KIR2DS3), an unconjugated antibody was used (Table 2). For its detection, the cells were incubated with the anti-KIR2DS3 antibody, washed, and incubated with the secondary antibody (polyclonal PE-conjugated F(ab′) anti-rabbit IgG from eBioscience, catalog #12-4739-89). Since antibodies specific for all KIRs were not available, combinations of two antibodies, each of which binds to a different subset of KIRs, were used as specified in Table 3. Controls were incubated under similar conditions with fluorochrome-conjugated isotype-matched control antibodies (all from eBioscience). For some receptors (e.g., KIR2DL4), which are expressed intracellularly in endosomes/lysosomes, the cells were fixed and permeabilized with Cytofix/Cytoperm reagents (BD Biosciences) before the addition of the marker-specific antibodies. After the addition of the antibodies, the reaction tubes were gently vortexed and incubated for 30 minutes at room temperature. The cells in each tube were washed twice with PBS/FBS, resuspended in 2% paraformaldehyde, and analyzed by flow cytometry using BD LSRII Fortessa. Single fluorochromes were used for compensation (elimination of spectral overlapping) and for the setting of gates using a minus one method. The data was acquired and analyzed using FACSDiva (BD Biosciences) and FlowJo (Tree Star).

The gates for lymphocytes were set by forward and side scatter (Supplementary [Supplementary-material supplementary-material-1]). Minus one fluorescence (MOF) was used to determine the expression of markers for NK cells. The MOF was set with the cocktail of fluorochrome-conjugated antibodies used to gate NK cells and to which the fluorochrome-conjugated control antibody has been added. The receptor-specific fluorochrome-conjugated antibody was added to the MOF cocktail and used to determine the expression of the NK cell marker in question as described [25]. The normalized MFIs were pooled and used for comparisons between patients and controls. Absolute numbers of cells per ml were determined using CountBright™ Absolute Counting Beads (Invitrogen, catalog #C36950) following the manufacturer's instructions.

### 2.3. Determination of NK Cells' Recent History of Degranulation/Cytotoxicity

The surface expression of CD107a, also known as LAMP-1 (lysosome-associated membrane protein-1), has been used as a measure of recent cytotoxic activity (degranulation) of NK cells. CD107a is normally expressed on the inner surface of cytotoxic granules in NK cells (and CD8^+^ T lymphocytes (CTL)). When the cells degranulate (upon mediating cytotoxicity and/or the release of cytokines), CD107a is translocated onto the cell surface and remains there for a few hours. It prevents NK cell/CTL death from their released cytotoxicity mediators [26]. Therefore, at any given time, the surface expression of this molecule in NK cells (and CTL) correlates with their activation status as well as with their recent degranulation events [27]. We determined CD107a expression on NK cells with and without prior stimulation with K562 cells, which are erythroleukemic cells that lack surface expression of MHC class I molecules and are often used as target cells to activate NK cells and determine their cytotoxicity [28, 29]. The assay was conducted as described [30]. Briefly, 100 *μ*l of whole blood was used with and without the addition of 1 × 10^5^ K562 cells at 37°C for one hour. Thereafter, FITC-conjugated anti-CD107a antibodies (eBioscience, catalog #11-1079-42) and the Golgi stop (monensin; BD Bioscience) were added, and the cells were further incubated for 3 hours. The microcultures were then placed on ice, and the cells were stained with PerCp efluor 710-conjugated anti-CD3 and PE-Cy7-conjugated anti-human CD56 and APC-conjugated anti-human CD16 antibodies (Table 2). After lysis of RBCs with the RBC lysis buffer (eBioscience; catalog #00-4300-54), the cells were analyzed by flow cytometry. The expression was determined on CD3^−^CD56^bright/dim^CD16^+^ NK cells using LSRII Fortessa. The data was acquired by FACSDiva (BD Biosciences) and analyzed with FlowJo (Tree Star).

### 2.4. Statistical Analyses

Data were expressed as median and interquartile ranges or as means ± SE. Differences between means were tested for significance using the Mann-Whitney test, and *p* values ≤ 0.05 were deemed significant. All analyses were performed with GraphPad Prism version 4 (GraphPad Software).

## 3. Results

### 3.1. NK Cell Subsets in CD Patients and Healthy Controls

**Figure 1:**
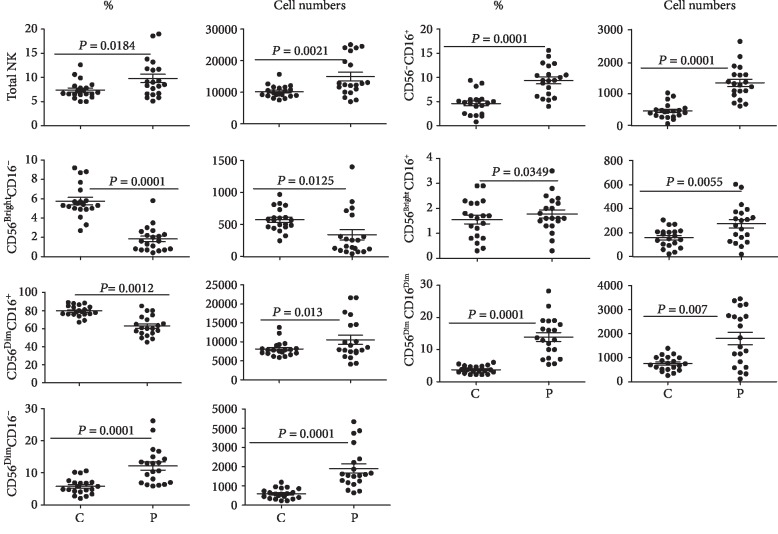
Percentages and absolute numbers of NK cells and their subsets. The figure shows percentages and absolute numbers (per ml) of the peripheral blood NK cells and their subsets (with respect to the expression of CD16 and CD56) in 20 CD patients (P) vis-à-vis 20 healthy control subjects (C). The figure shows means ± SE, individual data points, and *p* values.

We used flow cytometry to determine percentages and absolute numbers of the peripheral blood NK cells and their subsets based upon their expression of CD56 and CD16. Both percentages and absolute numbers of NK cells in the peripheral blood were significantly (*p* < 0.05) increased in patients' blood vis-à-vis healthy controls (Figure 1). Furthermore, significant perturbations were observed in different subsets in CD patients compared with the controls: significant decreases were observed in the percentages and numbers of the CD56^bright^CD16^−^ subset and a significant decrease was noted in the percentage of D56^dim^CD16^+^ subsets; however, a significant increase was observed in the absolute numbers of NK cells of this subset, and significant increases were observed in the percentages and absolute numbers of CD56^dim^CD16^−^ and CD56^−^CD16^+^ subsets.

The numbers of CD56^bright^CD16^+^ NK cells, but not their percentages, increased significantly (*p* < 0.05) in the patients' peripheral blood. Interestingly, we noted that a population of CD56^dim^ NK cells with relatively reduced expression of CD16 (called here as CD16^dim^ and shown in an arbitrarily set gate in Supplementary [Supplementary-material supplementary-material-1] (panel B, gate 4)) expanded in the patients' blood.

### 3.2. Expression of KIR on Peripheral Blood NK Cells from CD Patients and Healthy Controls

**Figure 2:**
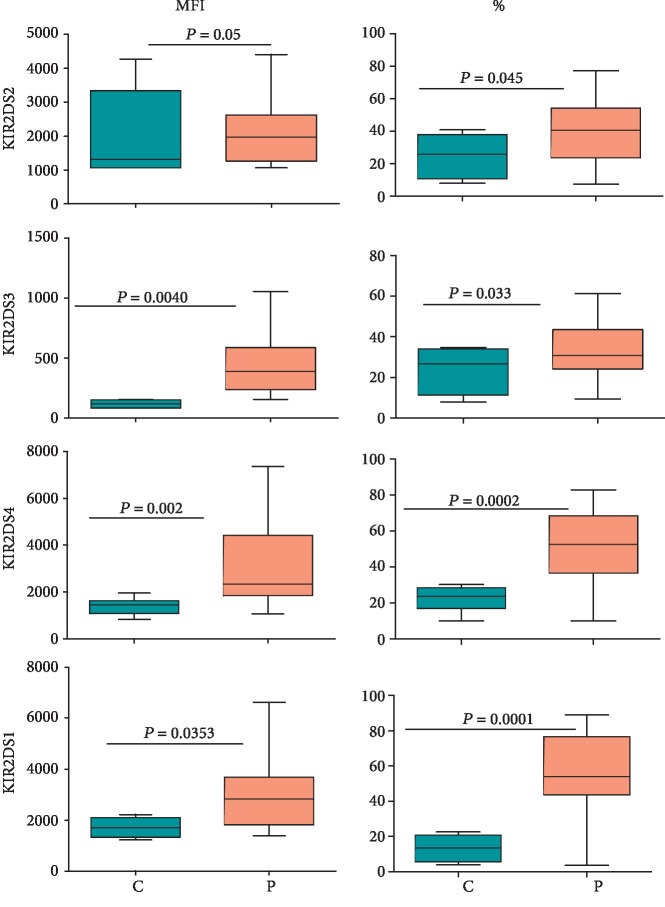
Expression of activating KIR on NK cells in CD patients and healthy controls. Fifty microliters of blood samples was used and stained for CD3, CD14, and CD19 and activating KIR (PE or FITC-conjugated) as described in Materials and Methods. The expression was determined on CD3-CD14-CD19-CD56^bright/dim^ (NK) cells. The figure shows mean fluorescence intensities (MFIs) and percentages of NK cells expressing the receptor from 15 treatment-naïve patients and 20 healthy controls. Each box and whisker plot indicates median, upper and lower values, interquartile range, and *p* values.

**Figure 3:**
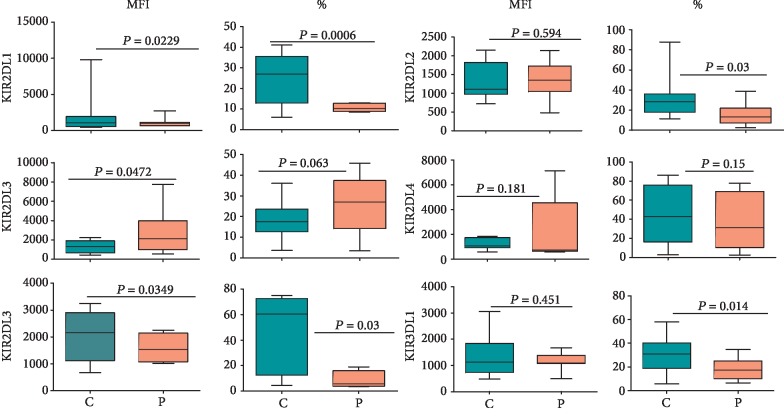
Expression of inhibitory KIR on NK cells in 15 treatment-naïve CD patients and 15 healthy controls. The cells were stained and gated as described in the legend of Figure 2 and stained for antibodies specific for inhibitory KIR as described in Materials and Methods. The figure shows median, upper and lower values, interquartile range, and *p* values.

The expression of KIR on NK cells in the peripheral blood was determined using KIR-specific antibodies as described in Materials and Methods. As shown in Figure 2, the expression of four activating KIRs, as determined by their mean fluorescence intensities (MFIs), was significantly increased in NK cells in CD patients compared with healthy controls. Furthermore, the percentages of NK cells expressing these receptors were also significantly increased in the patients except for KIR2DS3 for which the increase was statistically nonsignificant (*p* > 0.05). The combined staining for KIR2DS1/3/5 also showed their increased expression on the patient NK cells (Supplementary [Supplementary-material supplementary-material-1]). The expression of inhibitory KIR is shown in Figure 3. CD patients showed a general trend of a decreased expression of KIR2DL1, KIR2DL2, KIR2DL5, and KIR3DL1 on NK cells compared with those from healthy controls. Furthermore, the percentages of NK cells expressing these receptors also decreased significantly (*p* < 0.05) in these patients. The reverse was observed for KIR2DL3 for these parameters. Interestingly, the expression as well as percentages of NK cells expressing KIR2DL4, an atypical inhibitory KIR, showed nonsignificant (*p* > 0.05) changes between CD patients and healthy controls. Typical histograms for controls and patients for activating and inhibitory KIRs are shown in Supplementary Figures [Supplementary-material supplementary-material-1], respectively.

### 3.3. Expression of Non-KIR Receptors and Markers on NK Cells

**Figure 4:**
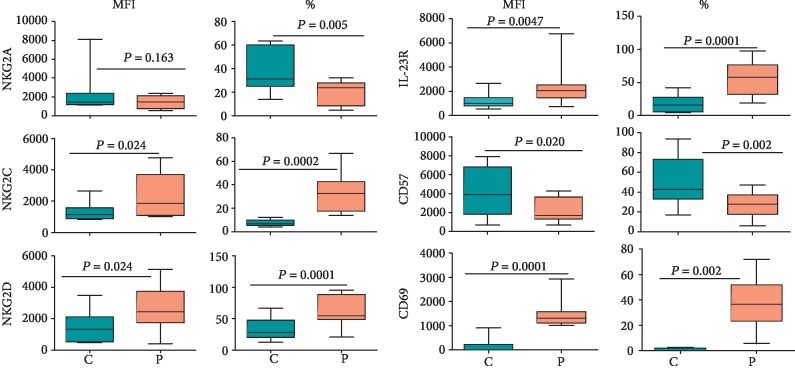
Expression of non-KIR markers on CD56^bright/dim^ NK cells in CD patients and healthy controls. The figure shows MFI and percentages of NK cells expressing the indicated marker in 15 treatment-naïve CD patients and 20 healthy controls. Each box and whisker plot indicates median, interquartile ranges, and *p* values.

We determined the expression of different non-KIR receptors on NK cells in the peripheral blood of CD patients and healthy control subjects. The expression of CD69 (an early marker of lymphocyte activation) [31], NKG2C (an activating receptor of the NKG2 family that binds to HLA-E [3]), NKG2D (an atypical member of the NKG2 family that binds to stress-induced proteins [3, 32]), and IL-23R (a component of the receptor complex that binds to IL-23 and whose genetic mutations have been associated with CD [33]) as well as the percentages of NK cells expressing these receptors was increased significantly (*p* < 0.05) in CD patients as compared with healthy control subjects (Figure 4). In contrast, although the expression of NKG2A (an inhibitory receptor of the NKG2 family that binds to HLA-E [3]) showed nonsignificant (*p* > 0.05) changes, the percentages of NK cells expressing this inhibitory receptor decreased significantly (*p* < 0.05) in the patients. The expression of CD57 (a sulfated carbohydrate epitope present on a variety of cell surface-expressed glycoproteins and a marker of NK cell terminal differentiation [34]) as well as percentages of CD57-positive NK cells showed significant decreases (*p* < 0.05) in the patients' blood compared to their counterpart cells from healthy controls (Figure 4). Typical histograms for the expression of these non-KIR receptors for CD patients and healthy controls are shown in Supplementary Figures [Supplementary-material supplementary-material-1].

### 3.4. Expression of Integrins on NK Cells

**Figure 5:**
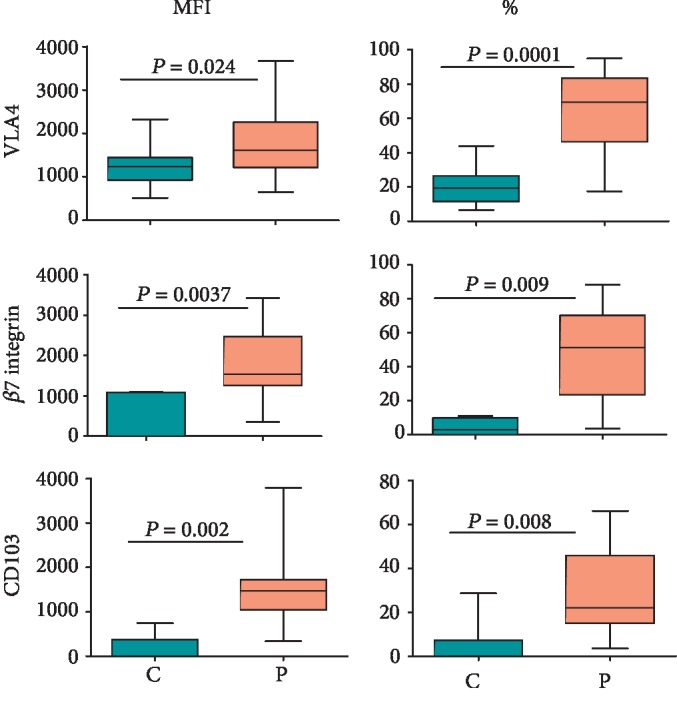
Expression of different integrins on CD56^bright/dim^ NK cells in CD patients and healthy controls. The figure shows MFI and percentages of NK cells expressing indicated integrins. Each box and whisker plot indicates median, interquartile ranges, and *p* values for 15 treatment-naïve CD patients and 20 healthy control donors.

Integrins are cell surface-expressed heterodimeric proteins (comprising *α* and *β* subunits) that mediate and sense cellular interactions with extracellular matrix proteins as well as cell-cell interactions. They play an important role in extravasation and homing of immune cells to different tissues [35]. We determined the expression of CD103 (*α*E*β*7), VLA4 (*α*4*β*1/CD49d-CD29), and integrin *β*7 on NK cells. The expression of these integrins was significantly (*p* < 0.05) increased on NK cells in CD patients. Furthermore, the percentages of NK cells expressing these integrins also tended to increase in the patients' blood (Figure 5). Typical histograms for the NK cell expression of these integrins in CD patients and healthy controls are shown in Supplementary [Supplementary-material supplementary-material-1].

### 3.5. Expression of CXC3R1, NKp46, and NKp44 on NK Cells

**Figure 6:**
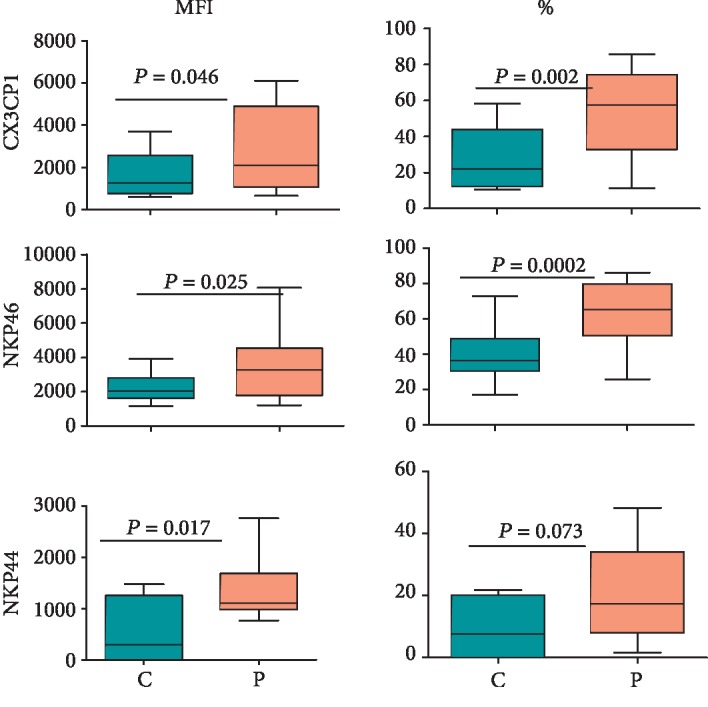
Expression of NKp46, NKp44, and CX3CR1 on CD^56bright/dim^ NK cells in CD patients and healthy controls. The figure shows MFI and percentages of NK cells expressing the markers. The box and whisker plots indicate median, interquartile ranges, and *p* values for 15 treatment-naïve CD patients and 15 healthy controls.

We also compared the expression of CX3CR1, NKp46, and NKp44 on NK cells between CD patients and healthy controls. CXC3R1 is a C-X3-C-type chemokine receptor that binds to CXC3L1/fractalkine and is implicated in the chemotaxis of immune cells to sites of inflammation in the body [36]. NKp46 and NKp44 are activating receptors belonging to the Natural Cytotoxicity Receptor (NCR) family [3]. The expression levels of CX3CR1 on NK cells tended to increase in CD patients compared with healthy controls; however, the increase was not significant (*p* = 0.128). Nevertheless, the percentages of NK cells expressing this chemokine receptor increased significantly (*p* < 0.05) in CD patients compared with the healthy controls (Figure 6). Furthermore, the expression as well percentages of NK cells expressing NKP46 and NKP44 increased significantly in CD patients compared with controls. Supplementary [Supplementary-material supplementary-material-1] shows typical histograms for the NK cell expression of these receptors.

### 3.6. NK Cells from CD Patients Show a History of Increased Recent Degranulation Events

**Figure 7:**
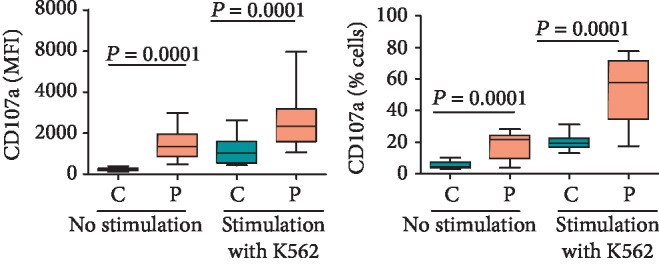
The surface expression of CD107a on CD^56bright/dim^ NK cells in CD patients vis-à-vis healthy controls with and without prior stimulation with K562. The panels show MFI of CD107a expression and percentages of CD107a expressed in NK cells. The box and whisker plots indicate median, interquartile ranges, and *p* values from ten treatment-naïve CD patients and the same number of healthy control donors.

The cell surface expression of CD107a (also known as lysosome-associated membrane protein-1 or LAMP-1) on cytotoxic cells (CD8+ T lymphocytes and NK cells) is a measure of their recent degranulation events as well as of their cytotoxic potential [37]. The level of expression of CD107a on the surface of unstimulated NK cells in CD patients was significantly (*p* < 0.05) higher compared with that in healthy controls (see MFIs in Figure 7). The expression was also higher in K562-stimulated NK cells of CD patients compared with similarly stimulated NK cells from healthy controls. Furthermore, significantly higher percentages of NK cells expressed this marker in CD patients with and without stimulation with K562 as compared with healthy controls (Figure 7).

### 3.7. Treatment Tends To Normalize NK Cell-Expressed Receptors, Integrins, and CD107a in CD Patients

**Figure 8:**
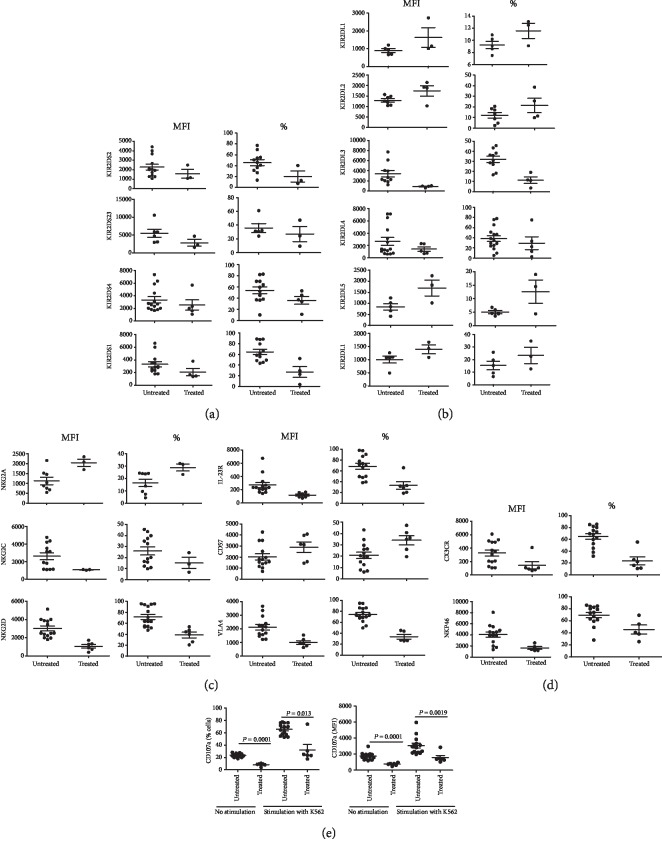
Comparison of NK cell expression of different parameters between treatment-naïve (untreated) and under-treatment (treated) CD patients. The figure shows NK cell expression of activating KIR (a), inhibitory KIR (b), non-KIR receptors and VLA-4 (c), NKP46 and CX3CR1 (d), and CD107a with and without stimulation with K562 cells (e). This figure shows means ± SE and *p* values.

In order to investigate the potential effect of treatment on the above-noted differences in NK cell expression of different receptors, integrins, and CD107a, we compared their expression between treatment-naïve and six under-treatment patients. However, due to insufficient number of PBMC, we could not investigate the expression of all the markers in all the six patients. As shown in Figure 8, the treatment tended to reverse the disease-induced changes in these NK cell parameters.

## 4. Discussion

In this study, we compared the expression of KIR, various non-KIR molecules, and integrins on peripheral blood NK cells from CD patients and their age-matched healthy control subjects. We also compared the history of recent degranulation events in these innate immune cells from the patients and healthy control subjects. Our results show increased percentages as well as absolute numbers of NK cells in CD patients. These increases were mainly due to increases in CD56^dim^CD16^−^, CD56^−^CD16^+^ and CD56^bright^CD16^+^ subsets. In contrast, these parameters were decreased for the CD56^bright^CD16^−^ subset. Interestingly, the percentages of CD56^dim^CD16^+^ NK cells decreased, but their absolute numbers increased in CD patients. It is noteworthy that this NK cell subset is the main cell type present in the peripheral blood and is highly cytotoxic [4]. Within this NK cell subset, the cells with lower expression of CD16 (designated here as CD56^dim^CD16^dim^ and shown in gate 4 in Supplementary [Supplementary-material supplementary-material-1] panel B) were noted in CD patients relative to the cells in healthy controls. Interestingly, this NK cell subset was found to be depleted in HIV-infected and immunodeficient individuals [38]. The expansion of this subset in CD patients may have resulted from a decreased expression of CD16 on NK cells. It is well known that CD16 is shed from the NK cell surface upon activation [39]. Recently, it was shown that the shedding may in fact increase NK cell potential to kill targets one after the other [40]. It is believed that immature NK cells express CD56 and they gradually reduce the expression of this marker and acquire CD16 as they mature and become cytotoxic [41]. The changes observed in different NK cell subsets in CD patients relevant to healthy controls could result from differences in proliferation and differentiation of these cells as well as from activation-induced shedding of CD16.

Our results suggest that the peripheral blood NK cells from CD patients possess higher cytotoxic potential as they express higher levels of CD107a constitutively as well as after their stimulation with NK-sensitive cancer cells (K562). It is noteworthy that NK cells that reside in, and/or infiltrate into, intestinal (and other) tissues are CD56^bright^CD16^-/low^ [42, 43]. These cells are less cytotoxic compared with the CD56^dim^CD16^+^ subset that is the predominant (90%) population in peripheral blood. However, the former subset becomes as cytotoxic as the latter one once it is exposed to proinflammatory cytokines such as IL-15 [4]. Given that increased levels of several proinflammatory cytokines have been described in the circulation as well as in the lamina propria of the gut in CD patients [44], it is very likely that NK cells in the gut of these patients also acquire high cytotoxic potential. In this regard, two studies showed increased numbers of NK cells with higher levels of perforin in the inflamed gut of CD patients although the results on their cytotoxic potential were contradictory [42, 43]. Confounding factors in these assays include variability in the disease activity of the patients (whether they had active disease or were in remission) and their treatment regimens. Several drugs prescribed for CD patients such as azathioprine, corticosteroids, and different biologics adversely affect the numbers and functions of NK cells in the body [45, 46]. In particular, azathioprine induces apoptosis in immature NK cells, inhibits proliferation of CD16^+^ NK cells, and reduces their cytotoxicity. It was demonstrated that CD16^+^ NK cells are increased in the lamina propria of colons in CD patients and they are highly cytotoxic. However, their numbers and cytotoxic potential decrease in the patients receiving azathioprine [46–48]. It is noteworthy that all the patients studied by Melgar et al. [42] showed no cytotoxicity of their lamina propria NK cells because all of them were receiving prednisolone or azathioprine. In contrast, we studied the cytotoxic potential in treatment-naïve patients.

We found increased expression of activating KIR and increased percentages of NK cells expressing these receptors. On the contrary, the expression and the percentages of NK cells expressing inhibitory KIR tended to decrease in CD patients compared with healthy controls. Increased percentages of NK cells expressing activating KIRs may result from increased frequencies of these genes occurring in CD patients [18]. Increased frequencies of activating *KIR* genes and of inhibitory *KIR* genes, which encode receptors with lower affinities for their MHC class I ligands, have been associated with several human autoimmune and chronic inflammatory conditions that include sepsis, ankylosing spondylitis, systemic lupus erythematous, leukemia, type 1 diabetes, and autism [49–54], reviewed in [15, 16]. In this regard, similar trends were observed in IBD patients by some researchers but not by others (reviewed in [55]). In individuals that harbor increased frequencies of activating *KIR* genes and/or of less inhibitory *KIR* genes, NK cells have a very low activation threshold. This means that their NK cells could be spuriously activated by environmental stimuli such as microbial infections, radiation, stress, and/or certain diets, cause autoaggression and produce more proinflammatory mediators.

We found that NK cells from CD patients expressed more CD69 compared with those from healthy controls. It is noteworthy that CD69 is a C-type lectin and is expressed on the cell surface as an s-s-linked homodimer. It is expressed very early on lymphocytes upon activation from different stimuli [31]. In NK cells, its expression correlates with that of CD107a [30]. CD69 acts as a costimulatory molecule, and its expression is associated with increased cytotoxicity and IFN-*γ* production from NK cells [56]. It is also known to suppress functioning of sphingosine 1 phosphate (S1P) receptor type 1 (S1P_1_) and promote lymphocyte retention in the thymus, lymph nodes, and inflamed tissues [56]. CD57 represents a marker of terminal differentiation for NK cells [34]. Its significantly (*p* < 0.05) decreased expression in NK cells in CD patients suggests that these cells have relatively increased proliferative capacity. This may explain increased percentages and absolute numbers of NK cells in these patients vis-à-vis healthy controls.

The IL23/IL-23R system has been implicated in the pathogenesis of CD. Loss-of-function variants of the IL-23R (a subunit of the receptor for IL-23, other being IL12R*β*1) are protective in humans from CD [33]. Increased expression of the receptor on NK cells observed here is in accordance with the results reported in another study [57]. The researchers also showed an increased expression of the cytokine (IL-23) in the circulation and inflamed colons of CD patients. It is noteworthy that a therapeutic monoclonal antibody, risankizumab, targets the p19 subunit of IL-23 and was clinically effective in active CD in a recent clinical trial [58].

In line with the results concerning the expression of activating and inhibitory KIRs, we found an increased expression of activating non-KIR receptors NKG2C and NKG2D but a decreased expression of NKG2A. NKG2C-positive NK (and T) cells are often expanded in humans infected with HCMV, although no viral ligand for the receptor has been recognized [59]. Production of proinflammatory cytokines such as IL-15, IL-12, IL-21, and IL-18, and activating KIR plays a role in this expansion. Interestingly, IL-21, a proinflammatory cytokine whose expression increases in the circulation of CD patients, induces the expression of NKG2C in immature NK cells [60, 61]. The expression of NKG2C is considered a biomarker for memory-type NK cells. It is believed that interactions between HLA-E, whose expression increases on the surface of HCMV-infected cells, and NKG2C cause expansion of NKG2C-positive cells in HCMV-infected individuals. NKG2C-positive NK cells show increased production of IFN-*γ* and TNF-*α* compared with NKG2C-negative NK cells. It is noteworthy that these cytokines have been implicated in the pathogenesis of CD [44]. The serostatus of our study participants with respect to HCMV is unknown. It is possible that a higher proportion of CD patients may be infected with this virus compared with healthy donors causing an increased expression of this receptor on their NK cells. The patient NK cells also express higher levels of NKG2D, an atypical member of the NKG2 family of NK cell receptors. Other than NK cells, activated human CTL also express this receptor. The receptor binds to the so-called “stress-induced” ligands such as MHC class I chain-related sequences (MIC) MICA and MICB and UL-16 Binding Protein- (ULBP-) 1-6 [32]. Normally, healthy cells in the body rarely express these ligands. It has been demonstrated that intestinal epithelial cells in CD patients express higher levels of these ligands as well as NKG2D on their NK cells [62] and hence may be targeted by these cells in this disease. Targeting NKG2D with a monoclonal antibody has shown some clinical benefit in IBD patients [63]. The expression of NKG2A, an inhibitory member of the NKG2 receptor family that binds to HLA-E, was observed to be decreased in CD patients. The receptor is expressed on developing NK cells before the expression of KIR [3, 10, 11]. Its decreased expression may contribute towards a reduced activating threshold of NK cells in CD patients.

The expression of different integrins such as CD103 (*α*E*β*7), VLA-4 (*α*4*β*1), and *β*7 was all increased on peripheral blood NK cells in CD patients compared to their expression on NK cells in healthy controls. These integrins play an important role in the extravasation and homing of NK cells (and other lymphocytes) into tissues [24]. CD103, also known as mucosal lymphocyte antigen-1, plays an important role in the homing and retention of lymphocytes in the gut [64]. It binds to E-cadherin expressed on the basolateral surface of intestinal epithelial cells. The integrin *α*4*β*7 is another gut-homing receptor that binds to Mucosal Addressin Cell Adhesion Molecule (MadCAM-1), a vascular adhesion molecule that directs lymphocyte extravasation to the intestinal lamina propria. Etrolizumab is a *β*7-specific therapeutic antibody that inhibits trafficking of lymphocytes to the gut by blocking both *α*4*β*7 and CD103. Another antibody, vedolizumab, targets the *α*4*β*7 heterodimer and is more specific. Both these antibodies are approved for therapy in IBD patients [65]. VLA-4 (*α*4*β*1; CD49d-CD29) binds to Vascular Cell Adhesion Molecule 1 (VCAM-1) expressed on activated vascular endothelial cells in inflamed tissues. It plays a role in the extravasation of lymphocytes into tissues. The humanized therapeutic monoclonal antibody natalizumab blocks this integrin by targeting its *α*4 subunit and has been approved for use in CD patients. However, it also blocks extravasation of lymphocytes into the brain through endothelial cells of the blood brain barrier, and its usage results in the development of Progressive Multifocal Leukoencephalopathy (PML) [66]. Our results suggest that increased expression of the integrins on peripheral blood NK cells in CD patients shows their increased potential to migrate to inflamed sites in the intestine.

The expression of NKp46, an activating receptor of the NCR family that is constitutively expressed on human NK cells, increased significantly (*p* < 0.05) in CD patients. The expression of this NCR (as well as certain other receptors such as NKG2D) is known to be significantly less on NK cells from children as compared with those from adults [67]. The expression of NKp44, another activating receptor of the NCR family, which is expressed only on cytokine-activated NK cells [3, 68], was also significantly increased in the peripheral blood NK cells of CD patients. Interestingly, Takayama et al. [69] reported that IFN-*γ*-producing NKp46-expressing CD3^−^CD56^+^ NK cells were increased and IL-22-producing NKp44-expressing CD3^−^CD56^+^ NK cells were decreased in the gut of CD patients. The NKp44^+^ NK cells in this study were ROR-C positive and most probably derived from LTi precursor cells and were not authentic NK cells.

CX3CR1, also known as the fractalkine receptor, binds to fractalkine, neurotactin, or CX3CL1 [70]. The receptor-positive lymphocytes are chemoattracted to inflamed sites in tissues. Interestingly, it has been documented that this chemokine receptor is involved in increased migration of T cells to the gut in IBD patients [71, 72]. The same may be true for NK cells. The increased expression of CX3CR1 on peripheral blood NK cells from CD patients suggests that they are more poised to migrate to the inflamed gut.

We found increased expression of CD107a on NK cells in CD patients compared to NK cells from healthy controls. The increased expression of this molecule was also observed on K562-stimulated NK cells from CD patients as compared with similarly stimulated NK cells from healthy controls. Other than lysosomes, CD107a is also expressed on the inner membranes of cytotoxic granules in NK cells and CTL [37]. When these cytotoxic cells undergo degranulation (upon triggering the killing of a target cell or upon secretion of cytokines), CD107a is translocated to the cell membrane and remains there for a few hours. Thus, at any given time, its level of expression on the surface of an NK cell reveals the cell's recent degranulation and cytotoxicity history [27]. A significantly increased expression of CD107a on peripheral blood NK cells from CD patients vis-à-vis healthy controls suggests that these cells in the patients had been more active than those in healthy controls with respect to their cytotoxicity and secretion of cytokines. Furthermore, the increased expression of CD107a in K562-stimulated NK cells in the patients compared with similarly stimulated NK cells from healthy controls suggests that the cells from the patients are more cytotoxic. To the best of our knowledge, no study has so far examined the expression of CD107a on NK (or other cytotoxic) cells in CD patients.

Our data on peripheral blood NK cells suggest that these cells are highly activated and more cytotoxic in CD patients when compared with those from healthy controls. An earlier study, however, reported that peripheral blood NK cells in CD patients show less than normal cytotoxicity due to the presence of an inhibitory factor in the circulation [73]. The authors showed that purified NK cells from the patients were quite normal in their cytotoxicity. More recently, it was shown that purified peripheral blood NK cells from CD patients were slightly more cytotoxic when used fresh. However, they were much more cytotoxic after their in vitro stimulation with IL-21 compared with similarly treated cells from healthy controls [61]. It is noteworthy that the concentrations of the cytokine are significantly increased in the gut and in the circulation of CD patients [74]. Thus, our results are in accordance with these observations. Our results show that treatment of CD patients with a variety of drugs and biologics tends to normalize the changes in the expression of NK cell-expressed receptors, integrins, and CD107a. The normalization of the NK cell activation and autoaggression may have contributed to the beneficial effects of the treatment in CD patients.

It may be relevant to ask whether the observed changes in CD patients in their peripheral blood NK cell compartment are a cause or consequence of the disease. Given that activating *KIR* genes are enriched in CD patients [18], it is tempting to speculate that these changes contribute towards causation of the disease. Therefore, novel drugs targeting NK cells may alleviate the disease severity in CD patients.

Taken together, our results suggest that increased activation status of peripheral blood NK cells in CD patients is a contributing factor to the pathogenesis of this chronic inflammatory disease. In addition to their increased cytotoxic potential, NK cells may also contribute to the disease in several other ways: increased production of proinflammatory cytokines (such as IFN-*γ* and TNF-*α*) and chemokines (such as CCR2 and RANTES), killing of Tregs, activated T cells and DC. Because of these multiple functions of NK cells, they have been postulated to play a dual role in autoimmune diseases [23, 75]. For example, by killing activated T cells, they could protect the host from T cell-mediated autoimmunity. However, their activation could also cause autoaggression and inflammation if the host's autologous cells become stressed/infected and become more susceptible to NK cell-mediated killing. Blocking of one or more NK cell activating receptors may alleviate disease progression as has been demonstrated in cardiac inflammation [76]. However, further studies are required to test the relevance of such an approach in CD patients.

Our study has some limitations. It was conducted using peripheral blood and not mononuclear cells from the lamina propria (mucosal NK cells) of CD patients. Furthermore, our NK cell gating may have included rare CD14^−^ nonclassical monocytes and innate lymphoid cells. Future studies should investigate whether mucosal NK cells obtained from the lamina propria of CD patients exhibit similar changes vis-à-vis their counterpart cells from healthy individuals. Furthermore, NK cells from the treated and untreated CD patients were used from limited numbers of the patients. They should be verified in larger cohorts of the patients.

## Figures and Tables

**Table 1:** Demographic and clinical parameters of CD patients.

Parameter	Patients	Controls
No.	21	20
Age	6-17	6-17
Sex^a^	15 : 5	14 : 6
Ethnicity	French Caucasian	French Caucasian
Disease activity	9 newly diagnosed	—
	6 relapsed	—
	6 in remission	—
Drugs	15 none	—
	6 under treatment	—

^a^Male : female ratio. The newly diagnosed patients had not received any treatment. The relapsed patients were previously treated with azathioprine, prednisone, anakinra, and natalizumab in different combinations. However, they were currently not receiving any treatment. The patients in remission were currently receiving TNF-*α* blocking antibodies (infliximab or adalimumab), prednisone, or azathioprine.

**Table 2:** List of antibodies used in this study.

Target	Antibody	Vendor	Cat #	Conjugated with
CD3	SK7	eBioscience	46-0036-42	PCp-eFluor-710
CD14	HCD14	BioLegend	325619	APC-Cy7
CD19	HIB19	BioLegend	302217	APC-Cy7
CD16	CB16	eBioscience	17-0168-41	APC
CD56	HD56	BioLegend	381310	APC
CD16	3G8	B&D	302016	PE-Cy7
2DL1	HP-3E4	B&D	556062	FITC
2DL1/2DS1	HP-MA4	BioLegend	339505	PE
2DL2/2DL3	DX27	BioLegend	312605	FITC
2DL3	180701	R&D	FAB2014F	PE/FITC
2DS3	—	LSBIO	LS-C165532	Unconjugated
2DL2/2DL3/2DS2	GL183	BC	IM2278U	PE
2DL4 (CD158d)	mAB33/(33)	BioLegend	347006	PE
2DS4 (CD158i)	JJC11.6	MB	130-092-680	PE
2DL5 (CD158f)	UP-R	BioLegend	341303	PE
3DL1	DX9	B&D	312706	FITC
3DL1/3DS1	Z27	BC	IM3292	PE
3DL2 (CD158k)	539304	R&D	FAB2878P	PE
NKG2A	131411	R&D	FAB1059	PE
NKG2C	134591	R&D	FAB138P	PE
NKp46	9E2	BioLegend	331907	PE
NKp44	P44-8.1	B&D	558563	PE
CD69	FN50	eBioscience	11-0699-42	FITC
VLA-4 (I*α*4/CD49d)	9F10	eBioscience	12-0499-42	PE
I*β*7	FIB27	BioLegend	121005	PE
CD103 (I*α*E)	BecACT8	BioLegend	350206	PE
IL-23R	218213	R&D	FAB14001P	PE
CD57	HCD57	BioLegend	322306	FITC
NKG2D	1D11	BioLegend	320805	PE
CXC3R1	2A9-1	eBioscience	12-6099-42	PE

B&D: Becton & Dickinson; LSBIO: Life Span Biotechnology; MB: Miltenyi Biotec; PCp: PerCP.

**Table 3:** Antibody combinations used to determine the expression of KIRs.

KIR	Antibody 1	Antibody 2
2DS1/S3/S5	(FITC)-HP-3E4 (2DL1)	(PE)-HP-MA4 (2DL1/2DS//S3/S5)
2DS2	(FITC)-DX27 (2DL2/3)	(PE)-GL183 (2DL2/3/2DS2)
2DL2	(FITC)-180701 (2DL3)	(PE)-GL183 (2DL2/3/2DS2)
3DS1	(FITC)-DX9 (3DL1)	(PE)-Z27 (3DL1/3DS1)

## Data Availability

All the data is provided within the manuscript.

## Supplementary Materials

Supplementary Materials Supplementary Figure 1: gating strategy for NK cells and their subsets. Supplementary Figure 2: expression of KIR2DS1/3/5 on NK cells in CD patients and healthy controls. Supplementary Figure 3: typical histograms for control (C) and patient (P) NK cells for the expression of activating KIR. Supplementary Figure 4: typical histograms for control (C) and patient (P) NK cells for inhibitory KIR expression. Supplementary Figure 5: typical histograms for control (C) and patient (P) NK cells for the expression of the indicated non-KIR receptors. Supplementary Figure 6: typical histograms for control (C) and CD patient (P) NK cells for the expression of NKG2 family receptors. Supplementary Figure 7: typical histograms for control (C) and CD patient (P) NK cells for their expression of integrins. Supplementary Figure 8: typical histograms for control (C) and CD patient (P) NK cells for the expression of NCR and CX3CR1.
