# Supplementary material for: Phenotypic and Functional Changes in Peripheral Blood Natural Killer Cells in Crohn Disease Patients
Source: Mediators Inflamm. 2020 Feb 19;2020:6401969. doi: 10.1155/2020/6401969 (PMC7049869; doi:10.1155/2020/6401969)
Supplement: Supplementary Materials — Supplementary Figure 1: gating strategy for NK cells and their subsets. Supplementary Figure 2: expression of KIR2DS1/3/5 on NK cells in CD patients and healthy controls. Supplementary Figure 3: typical histograms for control (C) and patient (P) NK cells for the expression of activating KIR. Supplementary Figure 4: typical histograms for control (C) and patient (P) NK cells for inhibitory KIR expression. Supplementary Figure 5: typical histograms for control (C) and patient (P) NK cells for the expression of the indicated non-KIR receptors. Supplementary Figure 6: typical histograms for control (C) and CD patient (P) NK cells for the expression of NKG2 family receptors. Supplementary Figure 7: typical histograms for control (C) and CD patient (P) NK cells for their expression of integrins. Supplementary Figure 8: typical histograms for control (C) and CD patient (P) NK cells for the expression of NCR and CX3CR1. [file 6401969.f1.pdf]

A

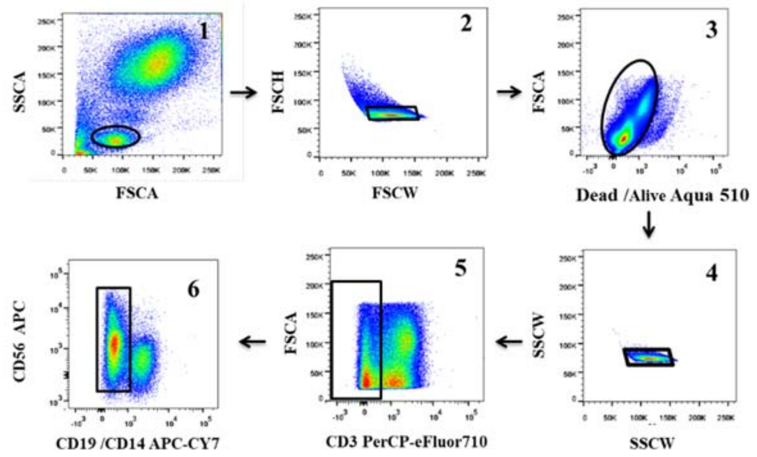


B

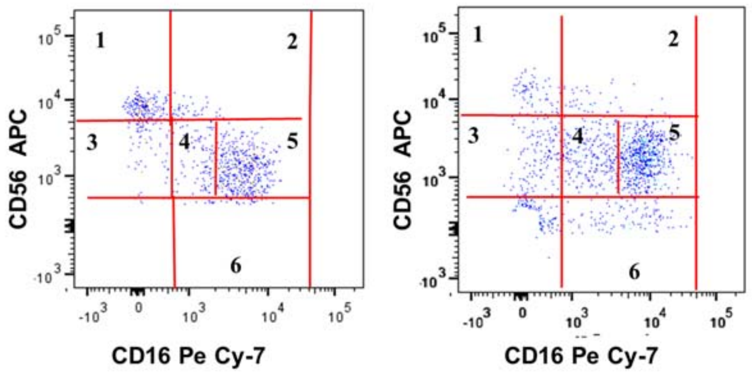


Supplementary Figure 1. Gating strategy for NK cells and their subsets. A. Lymphocytes were gated based upon FSC-A and SSC-A, doublets were eliminated by FSC-H and SSC-W, dead cells were eliminated using Aqua Blue and FSC-A, and doublets were again eliminated using FSC-H and SSC-W. Then CD3$^-$CD14$^-$CD19-CD56$^{+/-}$ cells were gated. B. CD3$^-$CD19$^-$CD14$^-$CD56$^{+/-}$ cells were further gated based upon the expression of CD16. The left and right panels show dot plots of NK cell subsets from a typical healthy control and a CD patient, respectively. 1: CD56$^{bright}$CD16$^-$, 2: CD56$^{bright}$CD16$^+$, 3: CD56$^{dim}$CD16$^-$, 4: CD56$^{dim}$CD16$^{dim}$, 5: CD56$^{dim}$CD16$^+$ and 6: CD56$^-$CD16$^+$.

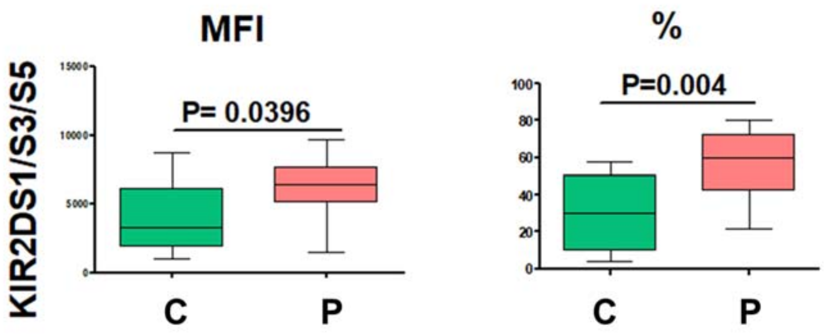


Supplementary Figure 2. Expression of activating KIR2DS1/S3/S5 on NK cells between treatment-naïve CD patients and healthy controls.

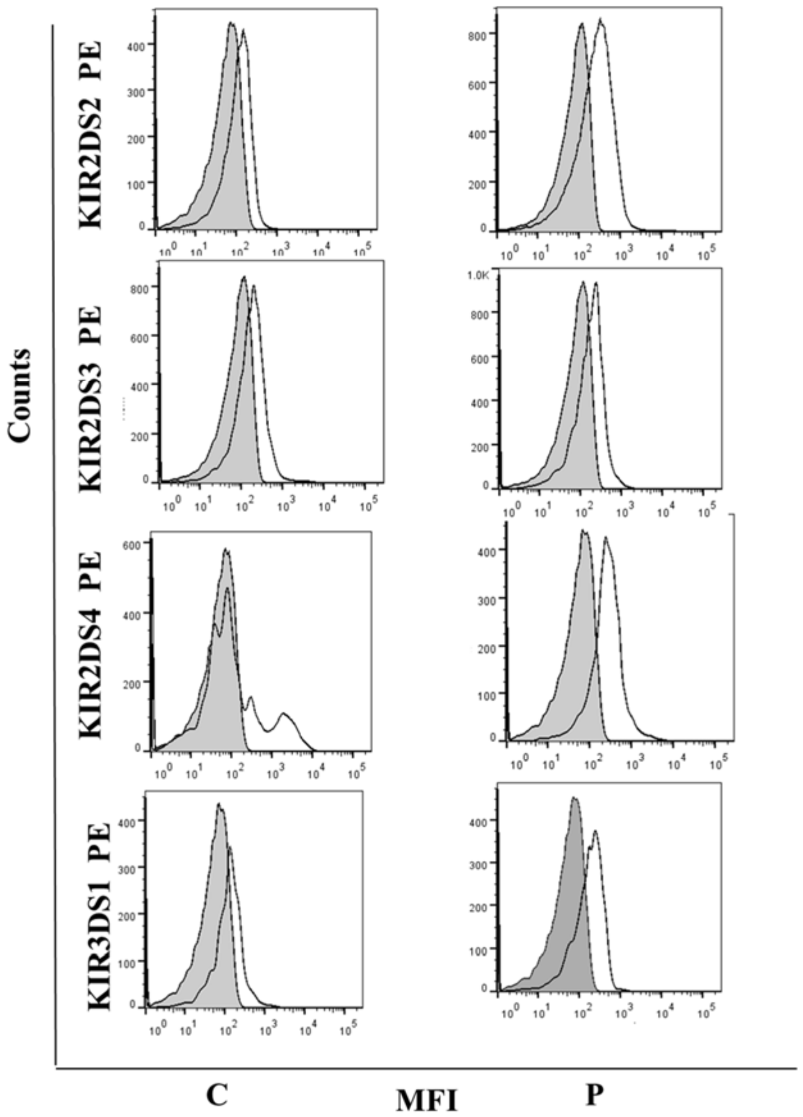


Supplementary Figure 3. Typical histograms for control (C) and patient (P) NK cells for the expression of activating KIR.

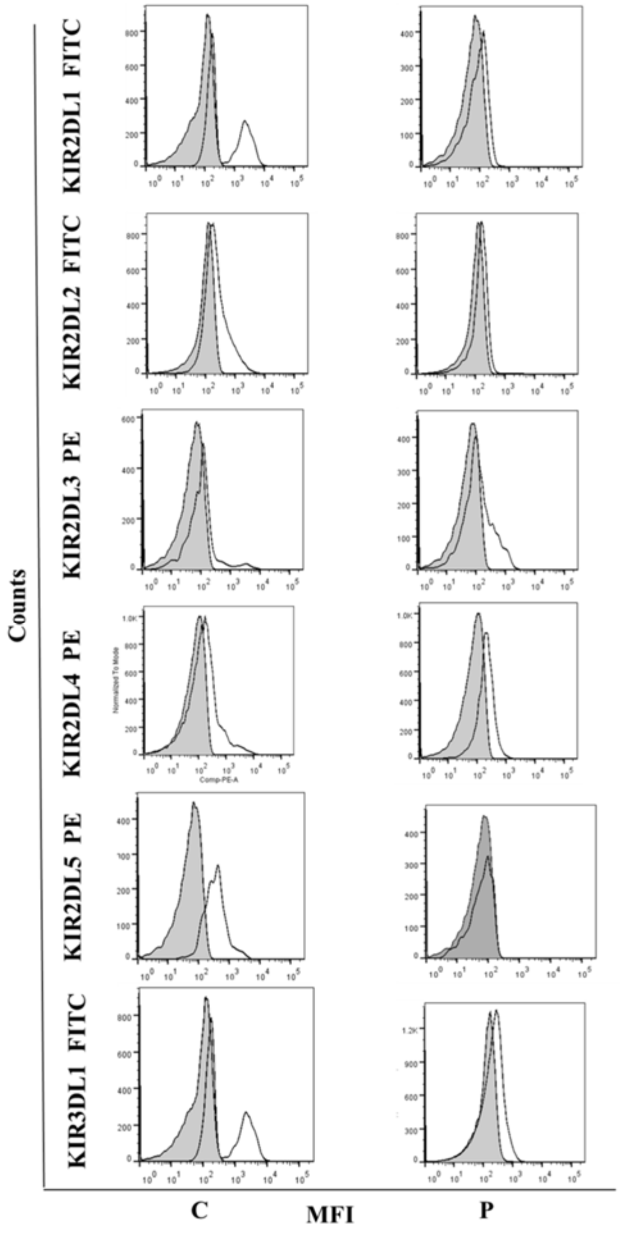


Supplementary Figure 4. Typical histograms for control (C) and patient (P) NK cells for the expression of inhibitory KIR.

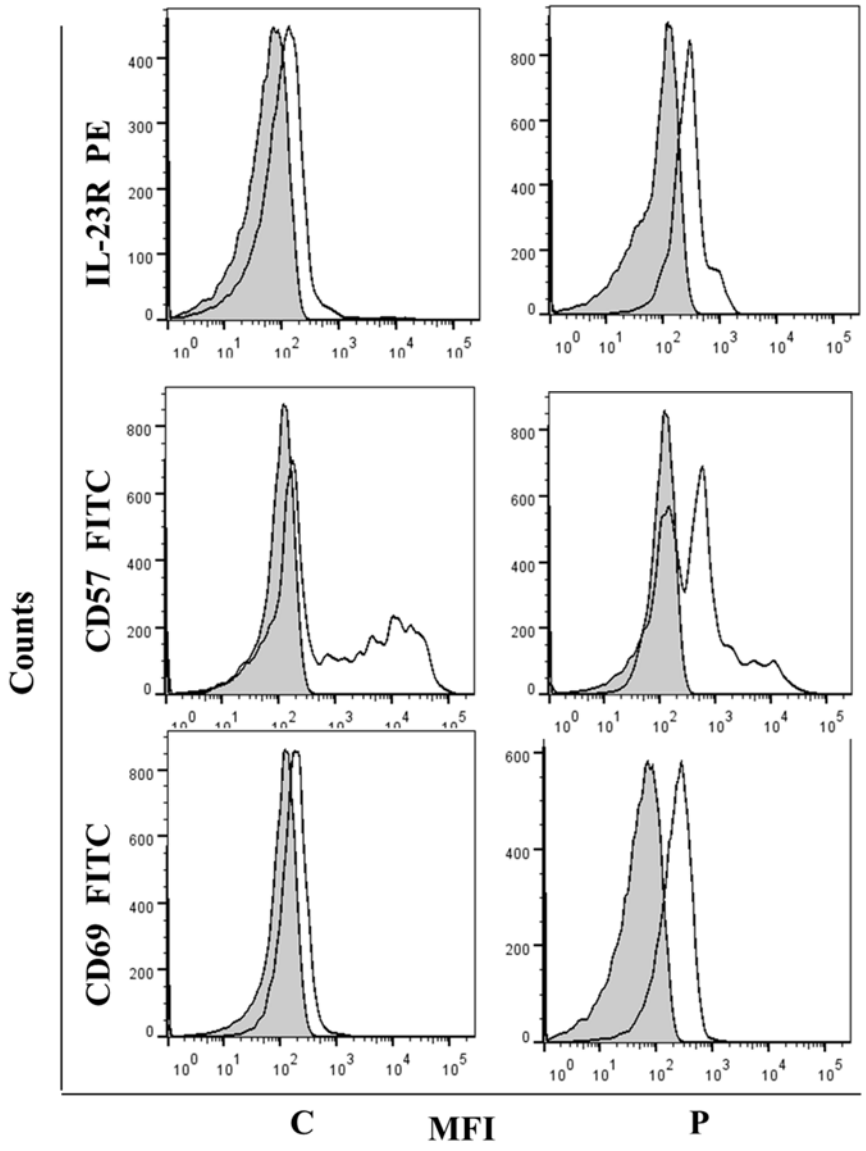


Supplementary Figure 5. Typical histograms for control (C) and patient (P) NK cells for the expression of indicated non-KIR receptors

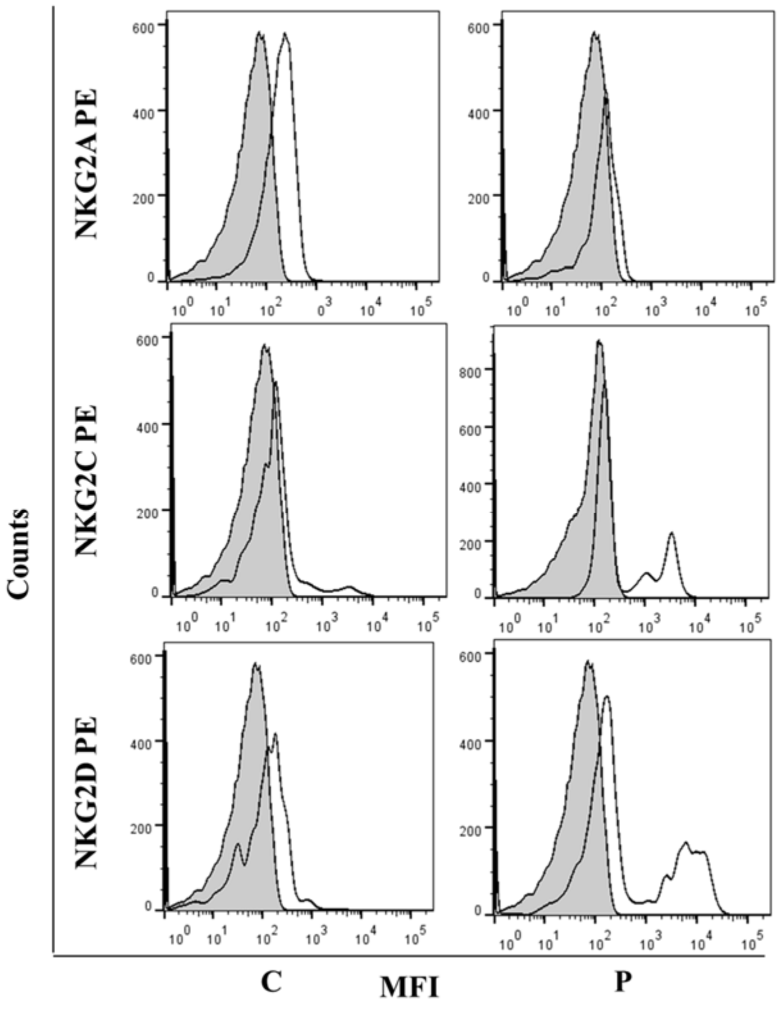


Supplementary Figure 6. Typical histograms for control (C) and CD patient (P) NK cells for the expression of indicated non-KIR receptors

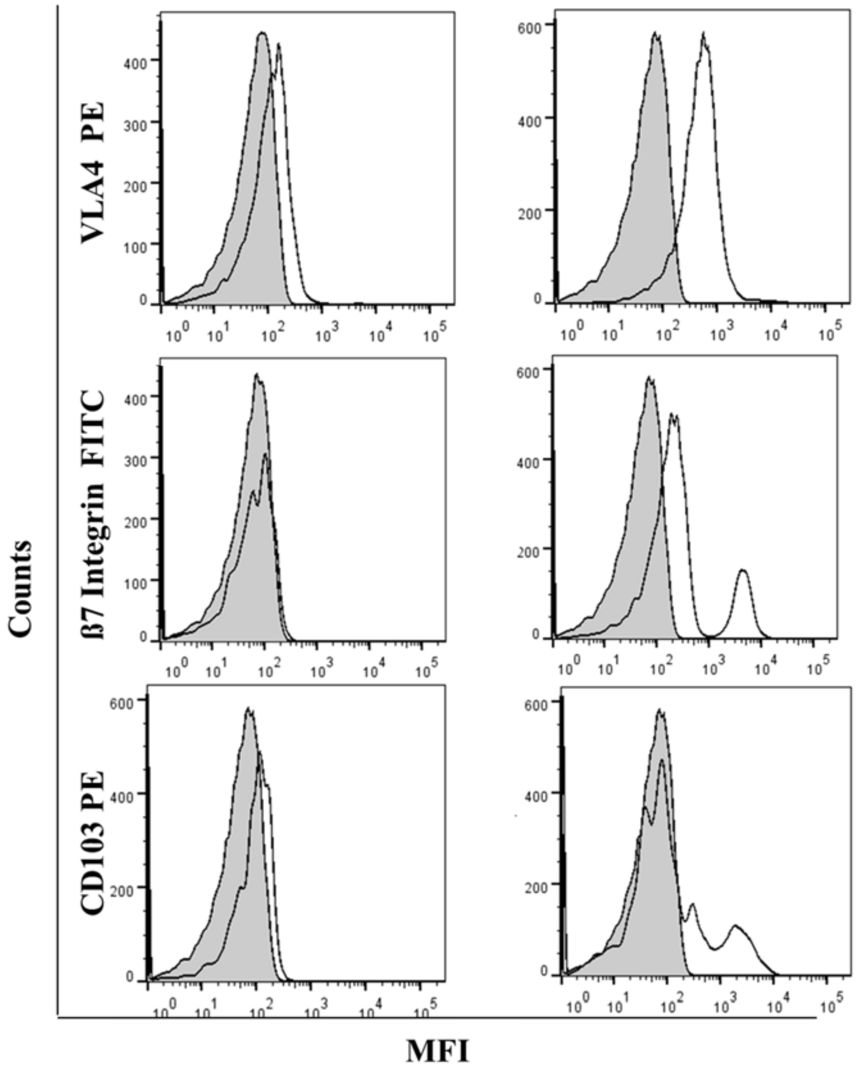


Supplementary Figure 7. Typical histograms for control (C) and CD patient (P) NK cells for their expression of integrins

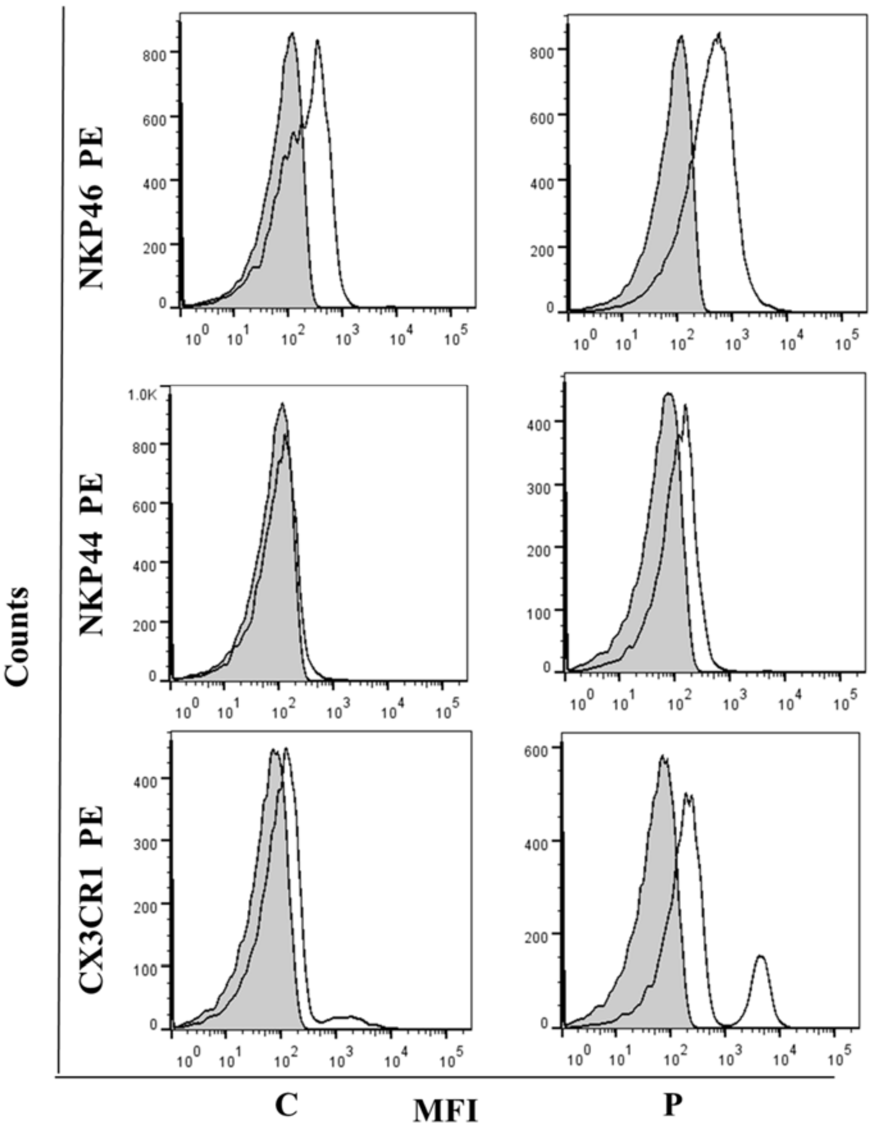


Supplementary Figure 8. Typical histograms for control (C) and patient (P) NK cells for the expression of NCR and CX3CR1.
